# Dual Antigen Display on an AP205 VLP Platform Elicits Potent and Durable Neutralization of EBV Infection in B Cells and Epithelial Cells In Vitro

**DOI:** 10.3390/vaccines14090735

**Published:** 2026-08-25

**Authors:** Xiaojuan Han, Ping Gao, Yuanyuan Shi, Chao Li, Xiaoyu Zhai, Guokai Feng, Musheng Zeng, Baidong Hou, Jian Song, Fuping Zhang

**Affiliations:** 1 CAS Key Laboratory of Pathogenic Microbiology and Immunology, Institute of Microbiology, Chinese Academy of Sciences (CAS), Beijing 100101, China; 2 College of Life Science, University of Chinese Academy of Sciences, Beijing 100049, China; 3 State Key Laboratory of Oncology in South China, Guangdong Key Laboratory of Nasopharyngeal Carcinoma Diagnosis and Therapy, Guangdong Provincial Clinical Research Center for Cancer, Sun Yat-sen University Cancer Center, Guangzhou 510060, China; 4 State Key Laboratory of Epigenetic Regulation and Intervention, Institute of Biophysics, Chinese Academy of Sciences, Beijing 100101, China; 5 Institute of Cardiovascular Sciences, Guangxi Academy of Medical Sciences, The People’s Hospital of Guangxi Zhuang Autonomous Region, Nanning 530021, China

**Keywords:** Epstein–Barr virus (EBV), virus-like particle (VLP), neutralizing antibodies, SpyTag/SpyCatcher, gH/gL and gB glycoproteins

## Abstract

Background/Objectives: Epstein–Barr virus (EBV) is a ubiquitous pathogen responsible for significant malignancies and autoimmune diseases, yet no prophylactic vaccine is available. The viral entry glycoproteins gL/gH and gB are essential for infection, but soluble forms are poorly immunogenic and fail to elicit durable neutralizing antibodies. Moreover, EBV infects both B cells and epithelial cells, demanding broad neutralization. This study aimed to develop a virus-like particle (VLP) platform that displays gL/gH and gB in a dense, repetitive array to overcome these barriers. Methods: We conjugated recombinant gL/gH and gB to Acinetobacter phage AP205 VLPs using SpyTag/SpyCatcher covalent linkage, generating monovalent and bivalent chimeric nanoparticles (co-displaying both antigens on the same particle). Mice were immunized with these VLP constructs or alum-adjuvanted soluble proteins, and antibody responses, neutralization titres against B-cell and epithelial-cell infection, as well as germinal centre responses and durability, were assessed over a four-month period. Results: AP205-conjugated nanoparticles elicited significantly higher antigen-specific IgG titres than soluble proteins. The chimeric VLP, co-displaying gL/gH and gB, induced the stronger neutralising antibodies, effectively blocking EBV entry into both B cells and epithelial cells. Mechanistically, VLP immunization drove robust and sustained germinal centre reactions, resulting in increased plasma and memory B cells, and maintained neutralising activity for at least four months. Conclusions: Precision nanoscale assembly of EBV entry glycoproteins on a synthetic VLP programs high-magnitude, broad-spectrum, and durable humoral immunity. The AP205-SpyTag platform offers a versatile and promising strategy for developing an effective prophylactic EBV vaccine.

## 1. Introduction

Epstein–Barr virus (EBV), a ubiquitous human gamma-herpesvirus with a seroprevalence exceeding 90% in adults globally, is a pathogen of profound medical significance. While primary infection often manifests as asymptomatic or as infectious mononucleosis, the virus establishes lifelong latency and is a well-established etiological agent for several malignancies, including Burkitt’s lymphoma, Hodgkin’s lymphoma, nasopharyngeal carcinoma, and a subset of gastric carcinomas [1,2,3,4,5]. Furthermore, compelling epidemiological and molecular evidence links EBV to the development of autoimmune disorders, most notably multiple sclerosis [6,7,8,9,10]. This extensive disease burden, combined with the near-universality of infection, underscores an urgent and unmet global need for a prophylactic EBV vaccine, a goal classified as a high priority by global health organizations.

A central challenge in designing such a vaccine is EBV’s complex biology, specifically its dual cellular tropism. The virus can infect both B lymphocytes, where it establishes latent infection, and epithelial cells, which support lytic viral replication and amplification. This dual tropism necessitates a vaccine strategy capable of blocking viral entry at both cellular portals to prevent initial infection and subsequent pathogenesis [11,12,13,14]. EBV viral entry is mediated by a coordinated glycoprotein machinery. Infection of B cells involves initial attachment via gp350 binding to CD21, followed by triggering of fusion by a complex of gL/gH with the accessory protein gp42. In contrast, epithelial cell entry is initiated by gL/gH binding directly to cellular integrins or ephrin receptors, independent of gp42 [15,16,17,18,19,20,21,22]. Crucially, in both pathways, the final, indispensable step of membrane fusion is executed by the conserved fusion glycoprotein B (gB). This makes gB, along with the essential gL/gH complex, a cornerstone target for comprehensive neutralization. While historical vaccine efforts have focused on the immunodominant gp350 to block B-cell infection, this strategy leaves the critical epithelial entry route unprotected. Therefore, simultaneous targeting of gL/gH and gB represents a rational approach to elicit antibodies that can block EBV infection comprehensively.

However, a significant barrier persists: recombinant soluble forms of gL/gH and gB are poorly immunogenic. When delivered with conventional adjuvants like alum, they typically fail to elicit the high-titer, high-affinity, and durable neutralizing antibodies required for effective, long-term protection [23,24,25]. This immunogenicity gap stems from the inability of soluble proteins to effectively cross-link B cell receptors (BCRs), a signal critical for robust activation, and their failure to efficiently stimulate T follicular helper cells needed for germinal center formation.

Over the past decade, several VLP-based approaches have been explored for EBV vaccination. For instance, Perez and colleagues (2017) generated insect cell-derived EBV VLPs incorporating gL/gH-EBNA1 or gB-LMP2 and demonstrated that these VLPs induced high neutralizing antibody titers and EBV-specific T-cell responses in immunized mice [24]. While these findings confirmed the potential of VLP-based strategies for EBV [24], several distinctions exist between their approach and ours. First, their VLPs were produced in eukaryotic (insect) cells and relied on native VLP assembly, whereas our platform is based on a well-defined, synthetic AP205 VLP scaffold produced in *E. coli* [26], allowing for greater modularity and precise control over antigen display. Second, our platform uses the SpyTag/SpyCatcher system for covalent, site-specific conjugation, ensuring stable and oriented antigen presentation—a feature not readily achievable with conventional VLP assembly [27]. Third, while their strategy incorporated intracellular EBNA1 and membrane-bound LMP2, our approach focuses exclusively on the key entry glycoproteins gL/gH and gB, which are the primary targets of virus-neutralizing antibodies. Finally, the AP205 carrier intrinsically encapsidates bacterial ssRNA, providing a built-in TLR7/8 adjuvant that promotes Th1-skewed responses, whereas insect cell-derived VLPs lack such an intrinsic adjuvant, necessitating external adjuvants for optimal immunogenicity [28,29].

Nanotechnology offers a powerful solution to this problem. Virus-like particles (VLPs) represent a premier vaccine platform that mimics the size, geometry, and repetitive surface architecture of native virions. This structural mimicry enables potent BCR cross-linking, dramatically enhancing B cell activation and antigen presentation [30,31,32]. The challenge lies in transitioning from biologically derived VLPs to truly programmable nano-immunogens that allow precise, stable, and modular antigen display [33,34,35]. Here, we address this challenge by engineering a synthetic VLP platform designed to deliver EBV gL/gH and gB as a potent, dual-target vaccine. We selected the *Acinetobacter* phage AP205 capsid as a robust, non-human, ~30 nm nanocage scaffold. This was functionalized using the SpyTag/SpyCatcher bioconjugation system, a genetically encoded peptide-protein pair that forms an isopeptide bond under mild conditions [36,37]. This system enables quantitative, site-specific, and covalent coupling of antigens, ensuring stable and oriented display in a high-density, repetitive array.

Beyond its structural function as an antigen display scaffold, the AP205 platform also serves as an intrinsic adjuvant. During expression in *E. coli*, the VLPs naturally encapsulate bacterial single-stranded RNA (ssRNA), which acts as a pathogen-associated molecular pattern (PAMP) that engages endosomal Toll-like receptor 7 (TLR7) and its adaptor protein MyD88. This engagement promotes dendritic cell maturation and drives a Th1-polarized immune response, counteracting the Th2-biased profile typically induced by alum adjuvants [34,35]. Of note, the AP205 platform is currently an investigational VLP carrier and has not yet received FDA approval for mass production of biologics. However, the immunological success of the AP205 platform has been demonstrated in multiple studies. For example, AP205-based vaccines have been shown to elicit potent neutralizing antibody responses against various pathogens, including HIV, influenza, and more recently SARS-CoV-2 [38,39,40,41,42]. Additionally, studies have confirmed that AP205 VLPs efficiently activate B cells and dendritic cells, induce robust germinal center responses, and promote long-lived plasma cell and memory B cell differentiation [43]. This self-adjuvanting property of the AP205 platform is a key distinguishing feature from earlier EBV VLP approaches, as it eliminates the need for external adjuvants while simultaneously promoting a more protective Th1-biased humoral response. In mice, AP205-conjugated EBV glycoprotein nanoparticles elicited substantially stronger antigen-specific antibody responses than traditional alum-adjuvanted soluble proteins. In addition, we constructed chimeric nanoparticles (Chimeric-AP205) that co-display gL/gH and gB on the same scaffold. This design elicited antigen-specific IgG titers substantially above those induced by the soluble protein controls, and more importantly, conferred durable, broadly neutralizing activity against both B-cell and epithelial-cell infection over a prolonged period.

Collectively, this study provides a versatile VLP-based strategy for EBV vaccination and illustrates a generalizable principle for applying synthetic nanotechnology in vaccine design.

## 2. Materials and Methods

### 2.1. Cell Lines

All cells were cultured under standard conditions (37 °C, 5% CO_2_). HEK293F cells (maintained in 293F medium, Union Biotech, Shanghai, China) were grown with orbital shaking at 120 rpm. For infection assays, Raji cells were cultured in RPMI1640 (Invitrogen, Waltham, MA, USA) supplemented with 10% FBS (GIBCO, Waltham, MA, USA), while HEK293T cells were maintained in DMEM (Invitrogen, Waltham, MA, USA) containing 10% FBS.

The Akata-EBV-GFP and CNE2-EBV-GFP cell lines were kindly provided by Professor Musheng Zeng’s laboratory at Sun Yat-sen University Cancer Center, as previously described [44,45]. Raji cells were a gift from Professor Xin Lin at Changping Laboratory, and HEK293F cells were obtained from Professor Baidong Hou’s laboratory at the Institute of Biophysics, Chinese Academy of Sciences.

### 2.2. Animals

All animals used in this work were maintained under specific-pathogen-free conditions following approval by the Animal Care and Use Committee of the Institute of Microbiology (HP-SQIMC-AS2024196), Chinese Academy of Sciences. BALB/c mice came from either in-house breeding or Charles River Laboratories (Beijing, China). For immunization, we used approximately equal numbers of 6- to 8-week-old males and females. Each mouse was considered as an independent experimental unit. The investigator performing the injections and the technician conducting the ELISA and flow cytometry analyses were blinded to the group allocation. Data analysis was also performed blinded by coding all samples prior to analysis. Upon arrival, all mice were allowed to acclimate to the animal facility for at least 3 days prior to the start of any experimental procedures.

### 2.3. Expression and Purification of EBV gL/gH and gB

The nucleotide sequences encoding EBV gL/gH and gB were designed based on the EBV strain B95-8 reference genome (GenBank: V01555.2) and synthesized for cloning into the pCEP4 vector (Addgene, Watertown, MA, USA). The full-length gH protein was truncated by removing the signal peptide, transmembrane domain, and cytoplasmic region. The truncated gH was connected to the full-length gL via a (G_4_S)_3_ linker, with SpyCatcher added at the C-terminus through a GSGG linker, and a His tag appended at the very C-terminus for purification. The full-length gB protein was truncated by removing the signal peptide and transmembrane domain, and the ectodomain (residues 23–683 aa) was selected. SpyCatcher was added at the N-terminus via a GSGG linker, and a His tag was inserted after the signal peptide at the N-terminus. For AviTag fusion constructs (gL/gH-AviTag and gB-AviTag), an AviTag sequence and a 6 × His tag were appended to the C terminus.

All four recombinant plasmids were transiently transfected into Expi293F cells (Thermo Fisher Scientific, Waltham, MA, USA) using PEI (Polyscience, Warrington, PA, USA) according to the manufacturer’s protocol. The secreted recombinant proteins were harvested from the culture supernatant and purified by Ni-NTA affinity chromatography (BBI Life Sciences, Shanghai, China). Purified proteins were analyzed by SDS-PAGE followed by Coomassie Blue (Sigma-Aldrich, St. Louis, MO, USA) staining, and protein concentrations were determined using NanoDrop spectrophotometry (Thermo Fisher Scientific, Waltham, MA, USA). 

### 2.4. Fluorescent Labeling of gL/gH and gB

A synthetic DNA fragment encoding BirA (Gene ID: 948469) (a biotin ligase used for enzymatic biotinylation) was cloned into pGEX (Addgene, Watertown, MA, USA). BL21(DE3) *E. coli* transformed with pGEX-BirA were grown in LB medium, and expression was induced with 0.1 mM IPTG (Sigma-Aldrich, St. Louis, MO, USA) at 18 °C for 18 h. His-BirA was purified on Ni-NTA agarose (BBI Life Sciences, China) following the manufacturer’s instructions. BirA is a biotin ligase enzyme that catalyzes the covalent attachment of biotin to a specific lysine residue within the AviTag sequence, enabling site-specific biotinylation of target proteins. Biotinylation of gL/gH-AviTag or gB-AviTag (100 mM each) was achieved by incubation with 1 mM His-BirA, 10 mM MgCl_2_, 10 mM ATP (Sigma-Aldrich, St. Louis, MO, USA), and 150 mM D-biotin (Sigma-Aldrich, St. Louis, MO, USA) at 30 °C for 1 h. The biotinylated products were then mixed with fluorochrome-conjugated streptavidin (BioLegend, San Diego, CA, USA) for 60 min at room temperature to yield fluorescent tetramers of gL/gH or gB. The fluorescent tetramers were stored at 4 °C in the dark in PBS containing 0.5% BSA and 0.05% sodium azide, and used within 1 week of preparation.

### 2.5. Vaccine Formulation

The AP205-SpyTag VLPs were a generous gift from Professor Baidong Hou’s laboratory at the Institute of Biophysics, Chinese Academy of Sciences [26,46]. For conjugation, 10 µg of gL/gH-SpyCatcher or gB-SpyCatcher was mixed with a six-fold molar excess of AP205-SpyTag in PBS and incubated on ice for at least 1 h to generate gL/gH-AP205 and gB-AP205 nanoparticles. For the chimeric vaccine, AP205 particles were conjugated with both gL/gH-SpyCatcher and gB-SpyCatcher at an equimolar ratio (5 µg of gL/gH-SpyCatcher + 5 µg gB-SpyCatcher) in PBS and incubated on ice for at least 1 h to generate Chimeric-AP205 nanoparticles. The purified VLP conjugates were stored at 4 °C in PBS containing 0.02% sodium azide and used within 1 weeks of preparation. For soluble protein controls, 10 µg of gL/gH or gB was adsorbed onto alum adjuvant (Imject Alum, Thermo Fisher Scientific, Waltham, MA, USA) at a dose of 100 µg of alum per mouse, according to the manufacturer’s recommendations.

### 2.6. Mouse Immunization

All animal experiments were conducted in accordance with institutional guidelines. A standard two-dose immunization schedule (prime at week 0, boost at week 3) was used for all experiments throughout this study, as illustrated in the corresponding experimental schematics. Unless otherwise specified, mice received a single dose of 10 µg of antigen (either VLP-conjugated or alum-adjuvanted soluble protein) via intraperitoneal (i.p.) injection. For alum-adjuvanted groups, 10 µg of soluble gL/gH or gB was adsorbed onto 100 µg of alum (Imject Alum, Thermo Fisher Scientific, USA) per mouse (delivered as 100 µL per injection) [46]. Serum samples were collected at week 2 (post-prime) for interim analysis and at week 4 (post-boost) for peak response analysis. For durability studies, additional serum samples were collected 4 months post-final boost for neutralization assay.

For route comparison, the same dose of each antigen was delivered via subcutaneous (s.c.) or intramuscular (i.m.) injection. For bivalent immunization studies, mice were randomly assigned to groups and immunized with one of the following formulations, each containing a total of 10 µg of antigen (measured as protein content): (1) cocktail soluble proteins (5 µg gB + 5 µg gL/gH) with alum; (2) chimeric nanoparticles (10 µg gL/gH/gB-AP205). For durability studies, mice were immunized with the same two-dose schedule (weeks 0 and 3) and serum samples were collected at weeks 4, 8, 12, and 16 post-prime.

Blood was collected via retro-orbital bleeding, and serum was separated by centrifugation at 3000× *g* for 10 min and stored at −20 °C until analysis.

### 2.7. ELISA for Antigen-Specific Antibody Detection

To quantify antigen-specific immunoglobulins present in serum samples, we coated microplates with purified gL/gH or gB lacking the SpyCatcher fusion domain (1 µg/mL in coating buffer). Serial dilutions of sera were then applied to the coated plates, and bound antibodies were detected using horseradish peroxidase (HRP)-conjugated secondary antibodies: anti-mouse IgG (Bethyl Laboratories, Montgomery, TX, USA), as well as anti-mouse IgG2a (Southern Biotech, Birmingham, AL, USA). The HRP substrate 3,3′,5,5′-tetramethylbenzidine (Sigma-Aldrich, St. Louis, MO, USA) was added, and the absorbance at 450 nm was recorded with a microplate reader (SpectraMax, Molecular Devices, San Jose, CA, USA). Antibody titers were defined as the reciprocal of the highest serum dilution yielding an optical density exceeding ten times the standard deviation measured from blank wells (without serum).

### 2.8. Flow Cytometry

Cells were resuspended in FACS buffer (PBS with 2% newborn calf serum, 2 mM EDTA, 0.1% NaN_3_) for surface staining. The staining panel comprised: PE-CF594 anti-B220 (RA3-6B2, BD Biosciences, Franklin Lakes, NJ, USA), allophycocyanin-Cy7 anti-CD19 (1D3, Invitrogen, Waltham, MA, USA), PE-Cy7 anti-IgM (II/41, Invitrogen), AF647 anti-IgD (11-26c.2a, BioLegend, San Diego, CA, USA), PE anti-GL-7 (GL7, BioLegend, San Diego, CA, USA), FITC anti-GL-7 (GL7, BioLegend, San Diego, CA, USA), AF700 anti-CD38 (90, BioLegend, San Diego, CA, USA), PerCP-Cy5.5 anti-CD8 (53-6.7, Invitrogen, Waltham, MA, USA), BV421 streptavidin (BioLegend, San Diego, CA, USA), PE streptavidin (BioLegend, San Diego, CA, USA). Flow cytometric data were collected on an LSR II (Becton Dickinson, Franklin Lakes, NJ, USA) and analyzed with FlowJo 10.8.1 (TreeStar, Ashland, OR, USA).

### 2.9. Neutralization Assay

Virus titers on different cell lines were determined before neutralization testing. For B-cell neutralization, 96-well plates with 50 μL of RPMI 1640 per well received serially diluted serum. Then 50 μL of CNE2-EBV-GFP (in RPMI 1640) was added to each well and incubated for 2 h at 37 °C. Subsequently, 100 μL of 10% FBS-RPMI 1640 containing 3 × 10^4^ Raji B cells were added per well, and incubation continued for 48 h at 37 °C. For epithelial cell neutralization, the same antibody-virus mixture was applied to wells containing 1 × 10^4^ 293T cells per well and incubated for 48 h at 37 °C. Flow cytometric assessment gave the percentage of GFP-positive cells. Negative controls were uninfected cells, while positive controls were cells infected with EBV alone. Neutralization percentage was expressed as [1 − (infection rate with antibody/infection rate of positive control)] × 100%. Non-linear regression analysis was performed to calculate IC_50_ values [45].

### 2.10. Negative-Stain Electron Microscopy

For negative-stain imaging of gL/gH-AP205 and gB-AP205, the proteins were diluted to ~0.02 mg/mL in PBS. Copper grids (300 mesh) coated with Formvar and carbon (Electron Microscopy China, Bejijing, China) were glow-discharged prior to use. A 5 μL drop of the sample was applied to each grid and incubated for 1 min, then blotted with Whatman No. 1 filter paper (GE Healthcare, Chicago, IL, USA). Staining was performed with 10 μL of 0.75% uranyl acetate (*w*/*v*) (Sigma-Aldrich, St. Louis, MO, USA) for 30 s, followed by removal of excess stain. Grids were viewed on an FEI Talos L120C G2 transmission electron microscope (Thermo Fisher Scientific, USA), and images were recorded with a Gatan Ceta 16M 4K × 4K CMOS camera (Gatan, Pleasanton, CA, USA).

### 2.11. Statistical Analysis

Sample sizes (*n*) varied across experiments and are specified in the figure legends. Results are presented as mean ± SEM. All statistical tests were performed on log-transformed titer values to satisfy the assumptions of normality and equal variance. For comparisons among more than two groups, one-way ANOVA with Tukey’s post-test was used. Each figure legend provides a detailed description of the statistical methods applied. Unpaired two-tailed Student’s *t*-test was used for comparisons between two groups. Statistical significance is reported as * *p* < 0.05, ** *p* < 0.01, *** *p* < 0.001, **** *p* < 0.0001; ns indicates *p* > 0.05. All analyses were carried out using GraphPad Prism 9.0.

## 3. Results

### 3.1. Design and Construction of EBV gL/gH-AP205 and gB-AP205 Nanoparticles

**Figure 1:**
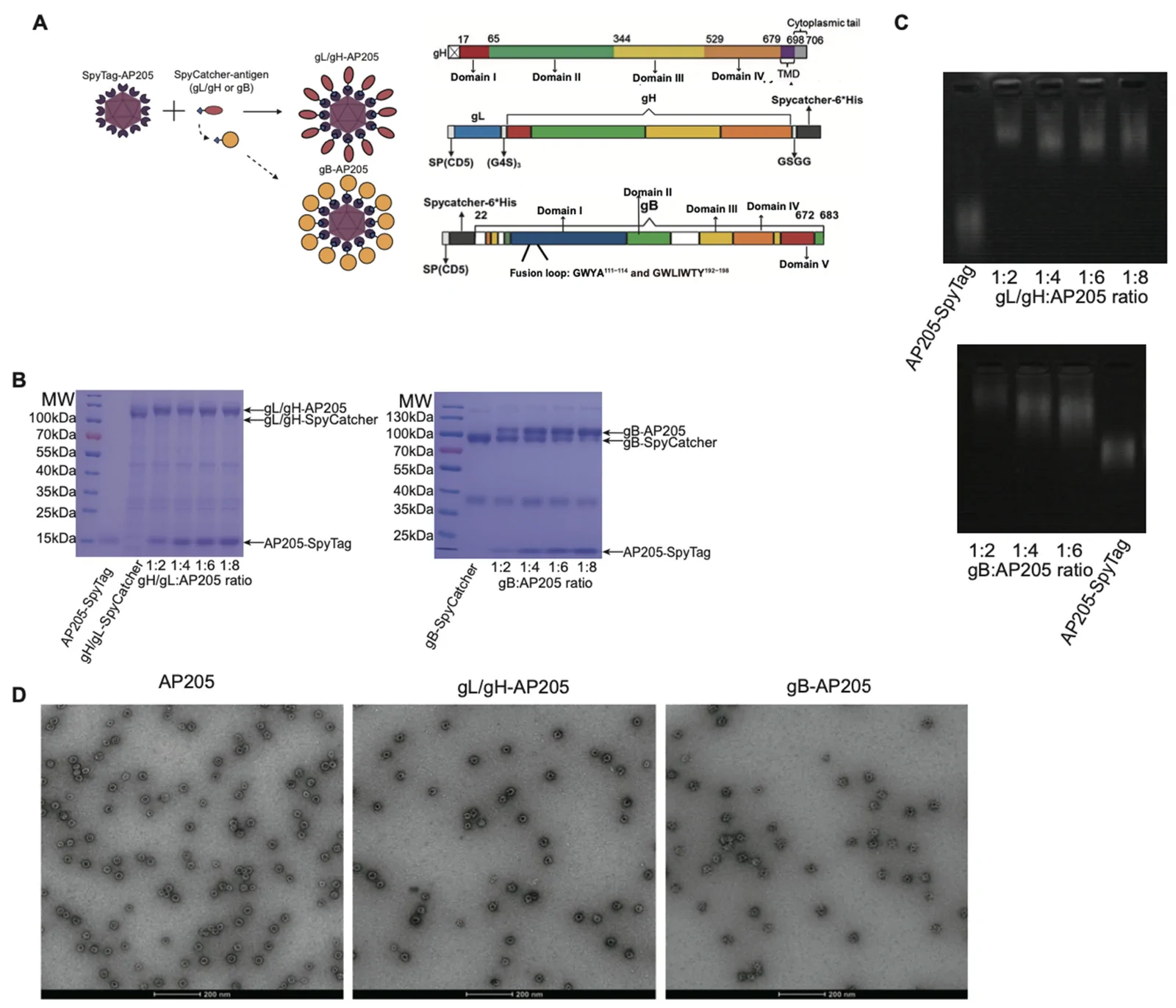
Molecular design and expression of EBV gL/gH- and gB-based AP205 VLP subunits. (**A**) Strategy for constructing gL/gH-AP205 and gB-AP205. AP205-SpyTag is combined with gL/gH-SpyCatcher or gB-SpyCatcher in vitro, allowing covalent bond formation via the SpyTag/SpyCatcher system. (**B**) SDS-PAGE gel showing gL/gH-SpyCatcher, gB-SpyCatcher and gL/gH-AP205, gB-AP205 particle. (**C**) Agarose gel showing gL/gH-SpyCatcher, gB-SpyCatcher and gL/gH-AP205, gB-AP205 particle. (**D**) Transmission EM micrographs of AP205-SpyTag, gL/gH-AP205, gB-AP205 particles.

To produce nanoparticle-based vaccine candidates targeting the essential EBV entry machinery, we anchored the viral glycoproteins gL/gH and gB onto a virus-like particle (VLP) scaffold. This was accomplished through the SpyTag/SpyCatcher system, which forms a spontaneous covalent bond. Specifically, recombinant fusion proteins comprising gL/gH-SpyCatcher and gB-SpyCatcher were engineered and subsequently coupled to AP205-SpyTag VLPs—particles assembled from the AP205 bacteriophage capsid protein that carries a genetic fusion with the SpyTag peptide (Figure 1A). During the conjugation reaction, the SpyCatcher-antigen fusions were supplied at a defined stoichiometry aimed at achieving roughly twenty antigen copies per particle, a density chosen to balance optimal antigen presentation with preservation of the VLP structure.

To construct an AP205-based EBV vaccine candidate, a particle composed of the fusion protein of the AP205-SpyTag (Figure 1A), and gL/gH, or gB-Spy-Catcher was added at approximately 20 molecules per AP205-SpyTag particle. The successful and efficient covalent conjugation of the antigens to the VLP scaffold was rigorously confirmed by SDS-PAGE and agarose gel analysis under reducing conditions (Figure 1B,C and Figure S1). The soluble gL/gH-SpyCatcher and gB-SpyCatcher proteins migrated at their expected molecular weights when analyzed alone. Upon conjugation to the AP205-SpyTag particles, a pronounced shift was observed. The majority of the antigenic material migrated at significantly higher molecular weights, consistent with formation of large complexes where multiple antigen subunits are covalently bound to the multimeric VLP scaffold (each AP205 particle comprises 180 capsid protein subunits). Critically, the near-complete disappearance of bands corresponding to the free, soluble antigen forms indicated a high coupling efficiency, with minimal unconjugated protein remaining in the final nanoparticle preparations. This analysis confirmed the formation of the desired products, which we designated gL/gH-AP205 and gB-AP205 NPs.

We next assessed whether the chemical conjugation process compromised the native architecture of the AP205 VLP platform. Transmission electron microscopy (TEM) of negatively stained samples provided direct visualization of particle morphology. The purified gL/gH-AP205 and gB-AP205 nanoparticles retained a uniform, spherical structure indistinguishable from the unconjugated AP205-SpyTag control particles. No evidence of aggregation, disintegration, or irregular shape formation was observed (Figure 1D), demonstrating that the SpyTag/SpyCatcher coupling chemistry is sufficiently gentle to preserve the structural integrity of the VLP core. This preservation of particulate morphology is a critical quality attribute, as the repetitive, high-density antigen array presented on a nano-scale particle is fundamental to its enhanced immunogenicity.

In summary, we successfully constructed two monovalent EBV nanoparticle vaccine candidates using a modular VLP platform. Biochemical and structural characterization confirmed the efficient and covalent display of gL/gH or gB antigens on intact, spherical AP205 particles, which also retained their immunostimulatory ssRNA payload. These well-defined NPs provide the necessary structural foundation for subsequent evaluation of their immunogenicity and neutralizing antibody induction.

### 3.2. Robust Immunogenicity of EBV gL/gH-AP205 and gB-AP205 Nanoparticles in Mice

**Figure 2:**
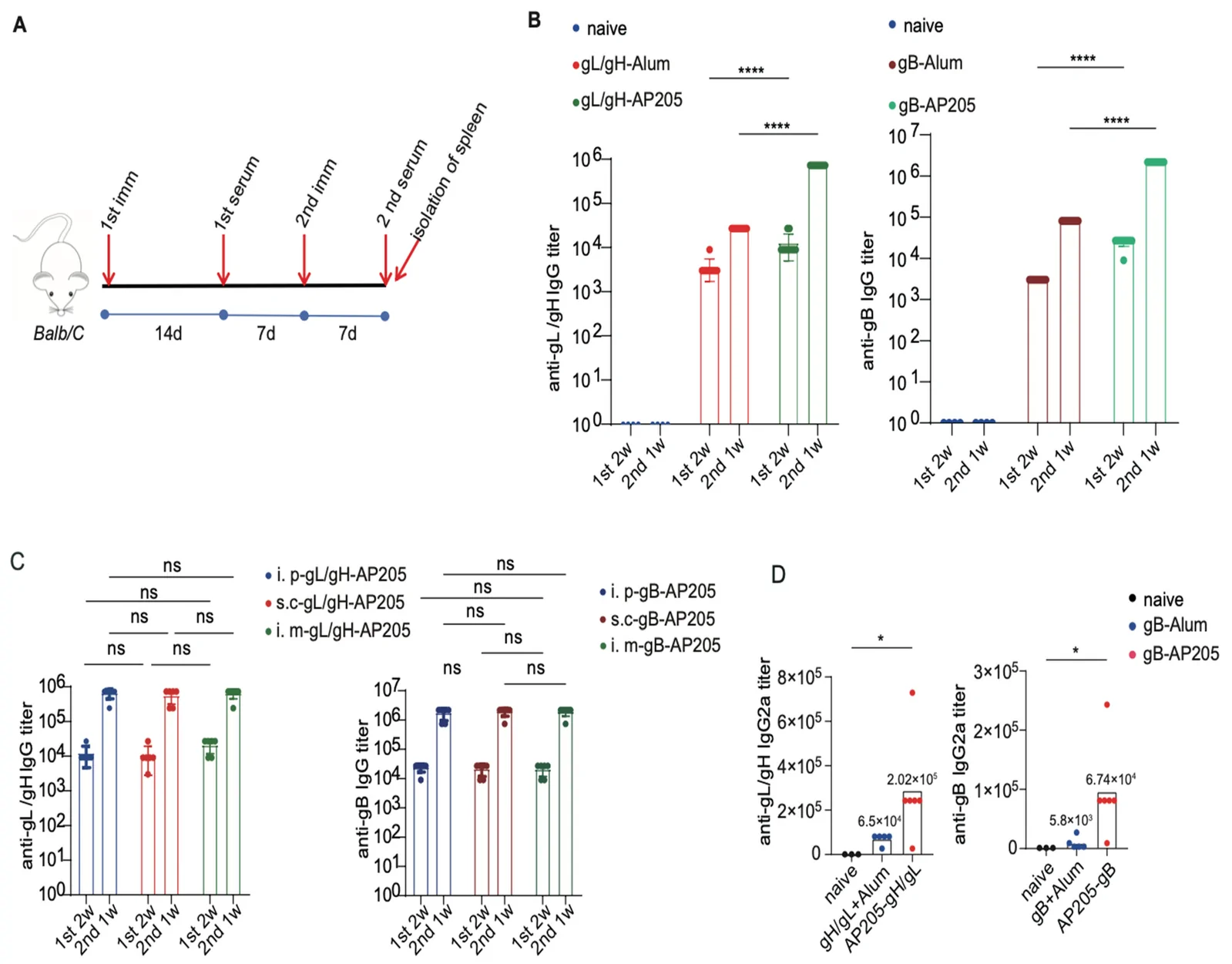
Immunogenicity of EBV gL/gH- and gB-based VLP subunits. (**A**) Schematic timeline indicating the first (1st) and second (2nd) immunization (imm) events and serum collection time points in mice (*n* = 5–6). (**B**) Antigen-specific serum IgG levels against gL/gH or gB were measured by ELISA in mice immunized with the indicated antigens. (**C**) ELISA quantification of serum anti-gL/gH or anti-gB IgG in mice that received the indicated antigens via intraperitoneal (i.p.), subcutaneous (s.c.), or intramuscular (i.m.) routes. (**D**) Serum titers of anti-gL/gH or anti-gB IgG2a isotypes in mice immunized with the indicated antigens. Endpoint titers are shown. Individual symbols represent data from single mice, and bars indicate the geometric mean for each group. Statistical comparisons of the virus titers were performed using Student’s *t*-test between groups immunized with gL/gH-AP205 or gB-AP205 and alum-immunized controls. (* *p* < 0.05, **** *p* < 0.0001, ns: not significant).

Having confirmed the structural integrity of the EBV nanoparticle constructs, we proceeded to evaluate their immunogenicity in a murine model. To benchmark the performance of our VLP display platform, we compared the immune responses induced by gL/gH-AP205 and gB-AP205 nanoparticles with those elicited by soluble gL/gH or gB proteins formulated with aluminum hydroxide (alum) adjuvant—a standard vaccine regimen [47,48,49,50]. BALB/c mice received two intraperitoneal injections on a prime-boost schedule at weeks 0 and 3, with serum collected at week 2 (post-prime) and week 4 (post-boost) for longitudinal tracking of antibody kinetics (Figure 2A).

Following the primary immunization, both nanoparticle and soluble protein regimens induced detectable antigen-specific IgG antibodies. However, a pronounced enhancement conferred by the VLP platform became evident after the booster dose (Figure 2B). In mice receiving gL/gH-AP205 or gB-AP205, anti-gL/gH and anti-gB IgG titers rose sharply—increasing by approximately 100-fold post-boost—and ultimately reached endpoint titers substantially higher than those achieved with alum-adjuvanted soluble proteins (Figure 2B). These results clearly demonstrate the superior immunogenicity of the nanoparticle format.

To assess the practical flexibility of our vaccine candidates, we next examined the impact of administration route. Mice were immunized with gL/gH-AP205 or gB-AP205 via subcutaneous (s.c.) or intramuscular (i.m.) injection, routes commonly used in human vaccination. Both alternative routes induced anti-glycoprotein IgG titers equivalent to those obtained via the intraperitoneal route (Figure 2C), confirming that the potent immunogenicity of the nanoparticles is independent of the injection method—a valuable attribute for translational development.

AP205-conjugated nanoparticles induced significantly stronger antigen-specific antibody responses than alum-adjuvanted soluble proteins, and this enhancement was observed across multiple immunization routes.

### 3.3. Immune Response Profiling in Immunized BALB/c Mice

To comprehensively characterize the quality of humoral immunity elicited by our vaccine candidates, we performed detailed serological profiling of immunized BALB/c mice. Antigen-specific IgG2a titers were measured by isotype-specific ELISA, as this subclass serves as a well-established serological surrogate for T-helper 1 (Th1)-polarized immune responses. This analysis allowed us to assess how vaccine formulation influences the resulting adaptive immune phenotype.

Quantification of serum IgG2a revealed that both soluble protein and nanoparticle vaccines induced detectable titers of this subclass. However, a marked divergence in immune polarization emerged between the two platforms: IgG2a levels were substantially elevated in mice immunized with gL/gH-AP205 or gB-AP205 nanoparticles compared to those receiving alum-adjuvanted soluble proteins (Figure 2D). This pronounced shift reflects a robust Th1-biased response triggered by the nanoparticle platform, consistent with the intrinsic adjuvant properties of the AP205 VLP scaffold. Its encapsulated ssRNA likely stimulates TLR7/MyD88 signaling, thereby promoting a cellular immune profile distinct from the Th2-skewed response typically associated with alum [46]. Notably, despite the dramatically higher overall antibody titers induced by the nanoparticles, the fundamental immunodominance hierarchy of the antigens remained unaltered. Collectively, this detailed profiling confirms that our VLP design not only amplifies antibody magnitude but also favorably redirects the immune response toward a Th1-dominant phenotype.

### 3.4. gL/gH-AP205 and gB-AP205 Vaccine-Elicited Antibodies Neutralize EBV Infection in B Cells and Epithelial Cells

**Figure 3:**
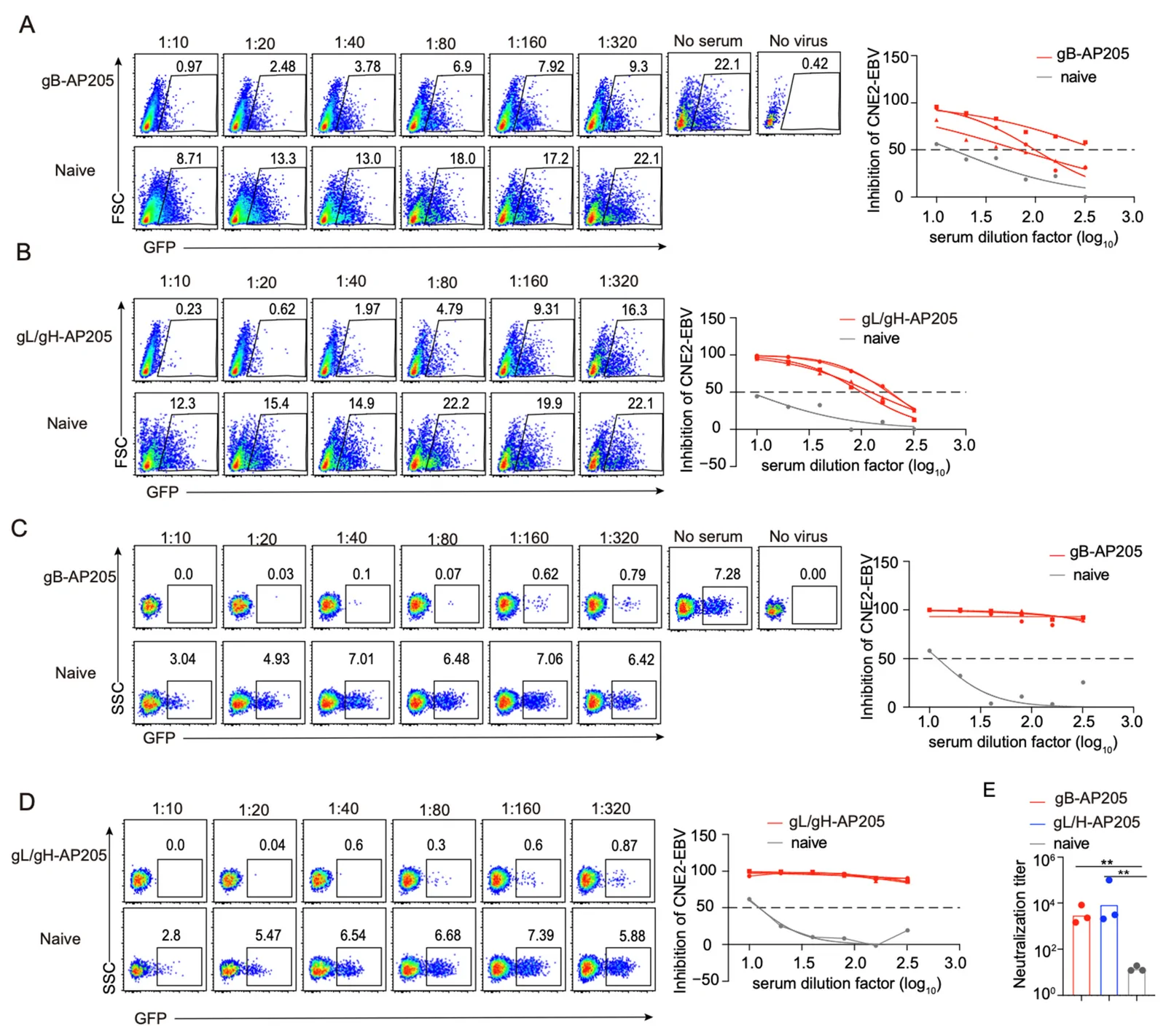
Neutralizing antibody responses elicited by EBV gL/gH- and gB-based VLPs protect B cells and epithelial cells from infection. Mice received a single dose of 10 µg of antigen-AP205 via intraperitoneal (i.p.) injection, administered twice with a three-week interval between doses. Then the serum was collected one week after the second immunization. (**A**,**B**) Flow cytometric assessment of the neutralizing activity present in with gB-AP205 (**A**), gL/gH-AP205 (**B**) immunized mice or naive mice sera collected after the second immunization, tested against EBV infection in Raji B cells. Individual symbols represent data from single mice (*n* = 3). (**C**–**E**) Flow cytometric assessment of the neutralizing activity present in with gB-AP205 (**C**), gL/gH-AP205 (**D**) immunized mice or naive mice sera collected after the second immunization, tested against EBV infection in 293T cells (*n* = 3). Curve fitting was used to determine the half-maximal inhibitory concentrations (IC50), which were presented as the neutralization titers in (**E**). one-way ANOVA with Tukey’s post-test was used. (** *p* < 0.01).

A key benchmark for a prophylactic EBV vaccine is its capacity to block infection in the virus’s two principal cellular targets—B cells and epithelial cells. We therefore assessed the functional neutralizing activity of sera from mice immunized with gL/gH-AP205 or gB-AP205 nanoparticles against both cell types. Sera from both vaccine groups exhibited potent and broad neutralizing activity, effectively preventing viral entry into B cells and thereby blocking the establishment of latency (Figure 3A,B). Importantly, this neutralizing capacity extended to epithelial cells, which play a central role in primary infection and viral shedding; both nanoparticle vaccines significantly reduced infection in these cells as well (Figure 3C–E). Collectively, immunization with gL/gH-AP205 and gB-AP205 nanoparticles generated high-quality antibodies capable of potently neutralizing EBV across its major cellular tropisms.

### 3.5. Chimeric gL/gH/gB-AP205 Formulations Are Highly Immunogenic and Elicit Potent Neutralizing Antibodies

**Figure 4:**
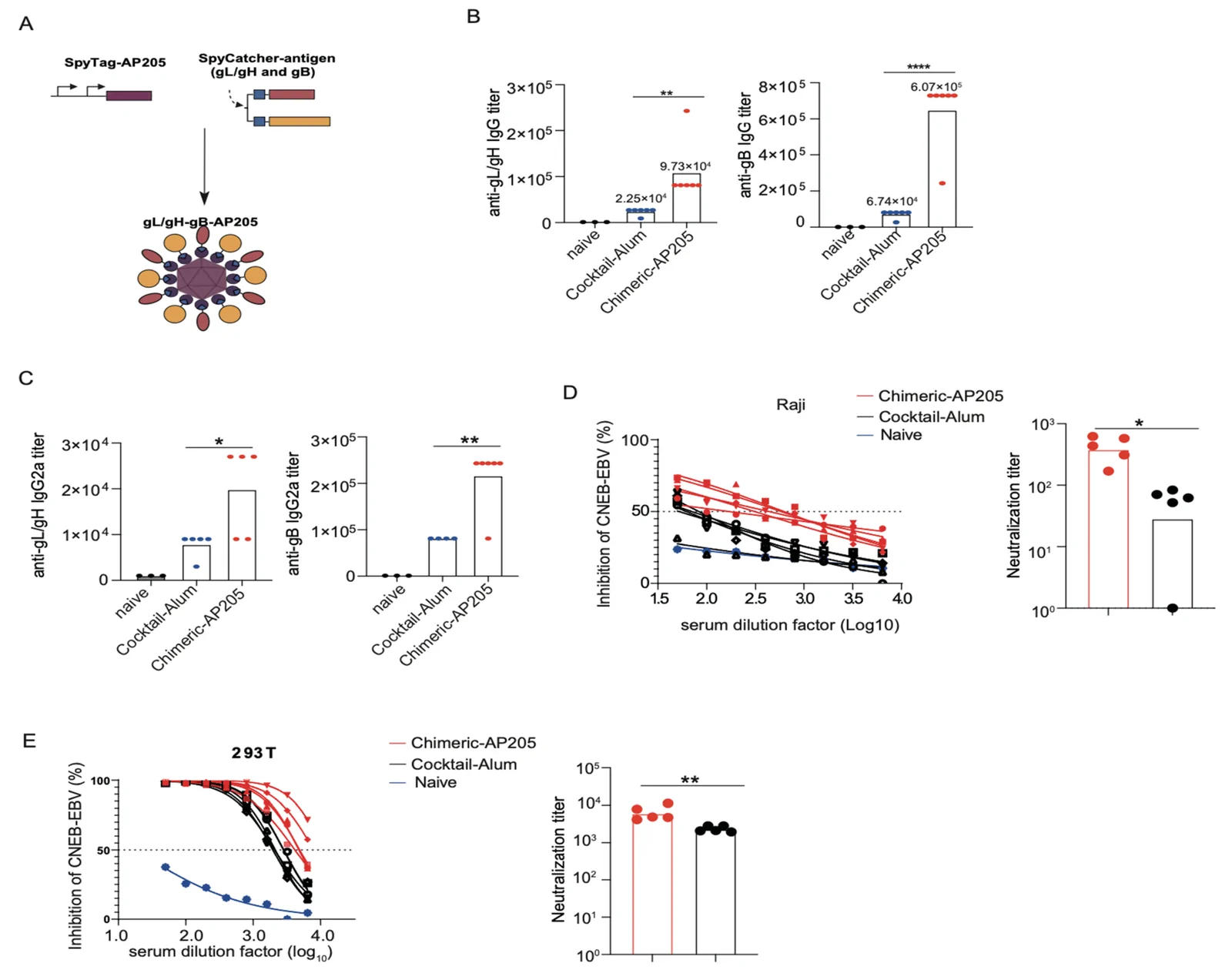
High immunogenicity and potent neutralization induced by chimeric gL/gH/gB-AP205 nanoparticle. (**A**) Strategy for constructing chimeric-AP205. AP205-SpyTag is combined with gL/gH-SpyCatcher and gB-SpyCatcher in vitro, allowing covalent bond formation via the SpyTag/SpyCatcher system. (**B**–**E**) Mice received a single dose of 10 µg of Chimeric-AP205 or cocktail-Alum via intraperitoneal (i.p.) injection, administered twice with a three-week interval between doses. Then the serum was collected one week after the second immunization (*n* = 4–6). (**B**,**C**) ELISA quantification of serum anti-gL/gH and anti-gB IgG (**B**), and IgG2a (**C**) in mice immunized with soluble EBV protein cocktail, or chimeric nanoparticle (Chimeric-AP205). (**D**,**E**) Neutralization titers (IC_50_) against EBV infection in Raji B cells (**D**) and 293T cells (**E**) were determined by flow cytometry using sera collected after the second immunization. Curve fitting was used to determine the half-maximal inhibitory concentrations (IC50) (**left**), which were presented as the neutralization titers in the (**right**). Student’s *t*-test was used (* *p* < 0.05, ** *p* < 0.01, **** *p* < 0.0001).

Given the encouraging results from monovalent immunizations, we next explored bivalent strategies that simultaneously engage both gL/gH and gB to better mimic the native antigenic complexity. Two formulations with identical antigen payloads were prepared and compared: a soluble protein cocktail formulated with alum (Cocktail-Alum), and a chimeric nanoparticle displaying both glycoproteins on the same VLP scaffold (Chimeric-AP205) (Figure 4A). BALB/c mice received two immunizations at weeks 0 and 3, with sera collected at week 4 for analysis.

The nanoparticle-based bivalent vaccines elicited markedly higher antigen-specific IgG titers than the soluble benchmark, as measured by ELISA against each individual antigen (Figure 4B). This advantage suggests that arranging both antigens on the same particle confers a substantial benefit in antigen presentation, leading to stronger B-cell activation and antibody production.

Assessment of antibody subclasses revealed that VLP formulations drove a robust Th1-polarized response, with elevated IgG2a levels, whereas the alum-adjuvanted cocktail remained Th2-dominant (Figure 4C). This profile is consistent with the intrinsic TLR7-stimulating property of the AP205 scaffold and was uniformly observed across all nanoparticle groups.

When we directly evaluated the neutralizing capacity of the bivalent sera against both relevant cell types, the VLP vaccines demonstrated potent blockade of viral entry into B cells and epithelial cells (Figure 4D,E). In both cases, neutralizing activity far exceeded that of the alum control, with the chimeric formulation again exhibiting the highest inhibitory titers. Collectively, these findings establish that bivalent nanoparticle vaccines—particularly the single-particle chimeric design—are highly effective at eliciting broadly reactive and durable neutralizing antibodies, and they confirm that the spatial organization of antigens on the VLP surface critically influences functional outcomes.

### 3.6. AP205-Based EBV Vaccines Elicit Potent Antigen-Specific Germinal Center Responses in BALB/c Mice

To elucidate the cellular mechanisms underlying the robust and durable humoral immunity induced by our nanoparticle vaccines, we investigated their ability to stimulate germinal center (GC) reactions. GCs are transient microanatomical structures within secondary lymphoid organs where activated B cells undergo clonal expansion, somatic hypermutation, and affinity-based selection, ultimately giving rise to high-affinity antibody-secreting plasma cells and long-lived memory B cells. Accordingly, the magnitude, quality, and duration of the GC response serve as fundamental predictors of vaccine efficacy.

**Figure 5:**
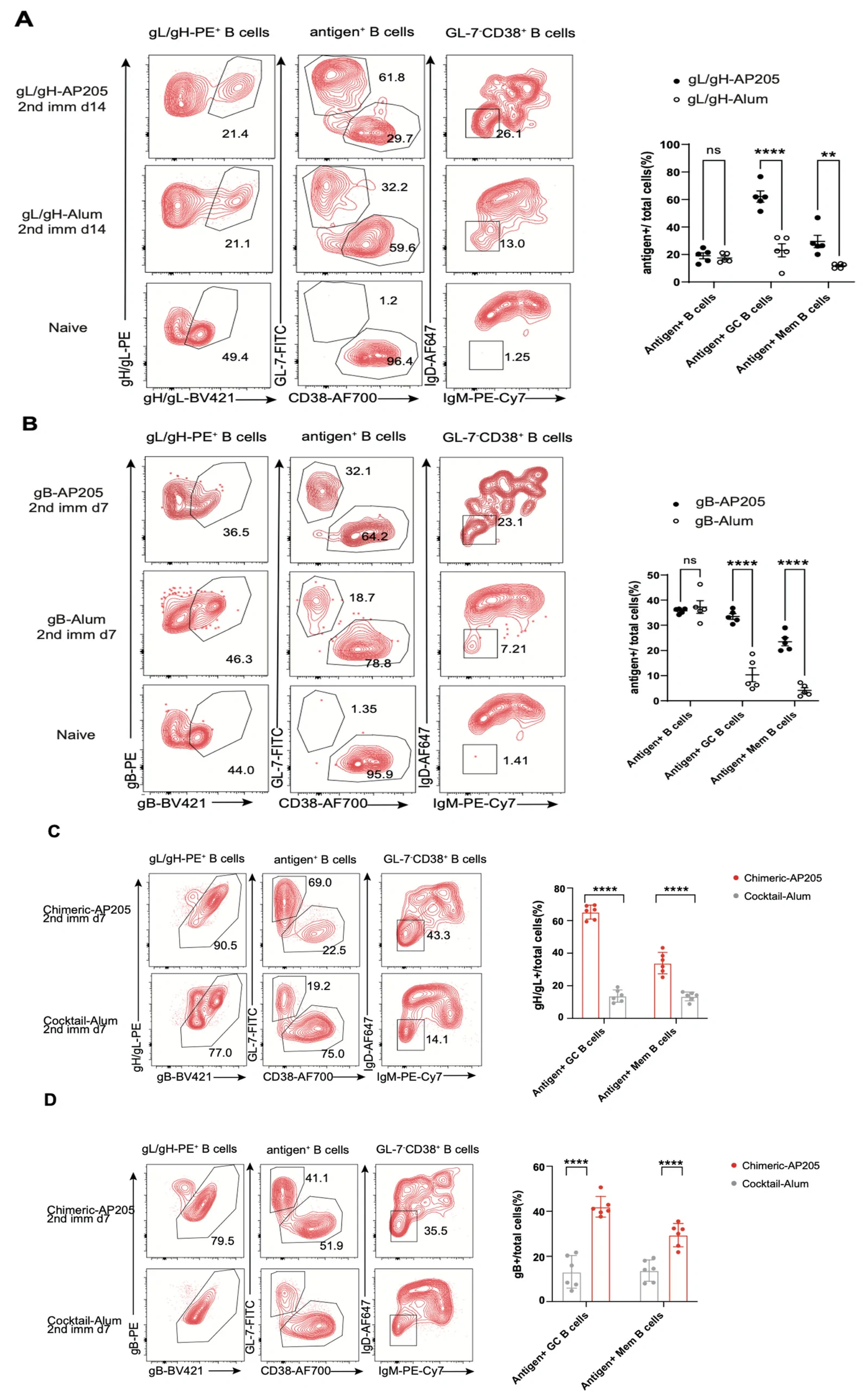
Antigen-specific GC responses elicited by EBV gL/gH- and gB-based VLP vaccines. Mice received a single dose of 10 µg of either antigen-AP205, or antigen-Alum via intraperitoneal (i.p.) injection, administered twice with a three-week interval between doses. Then the spleens were analyzed one week after the second immunization. (**A**–**D**) Detection of antigen-specific splenocytes using dual-color staining: (**A**) gL/gH-BV421/gL/gH-PE after immunization with gL/gH-AP205 or gL/gH-Alum; (**B**) gB-BV421/gB-PE after gB-AP205 or gB-Alum (*n* = 5); (**C**,**D**) gL/gH- or gB-based probes after Chimeric-AP205, or cocktail-Alum. Double-positive cells are defined as antigen^+^. In immunized mice, antigen-specific GC B cells (CD19^+^B220^+^GL7^+^CD38^−^), and switched memory B cells (swIg MemB: GL7^−^CD38^+^IgD^−^IgM^−^) were analyzed (*n* = 6). Numbers denote percentages relative to the parent gate. Each symbol represents one mouse; bars show geometric means per group. Student’s *t*-test was used (** *p* < 0.01, **** *p* < 0.0001, ns: not significant).

Using fluorophore-conjugated recombinant gL/gH and gB proteins as sensitive probes, we employed flow cytometry to track the induction and specificity of B-cell responses in the spleens of immunized BALB/c mice. Both our AP205 nanoparticle vaccines and the traditional alum-adjuvanted soluble protein controls induced a classical T-cell-dependent GC response, confirming antigen immunogenicity. In mice receiving gL/gH-AP205 or gB-AP205 vaccines, we detected significant populations of antigen-specific plasma cells (PCs; CD138^+^GL7^lo^), GC B cells (B220^+^CD95^+^GL7^+^), and isotype-switched memory B cells (swIg MemB; B220^+^CD38^+^IgD^−^IgM^−^). Spleens from naïve mice exhibited only background-level binding of the fluorescent probes to IgM^+^/IgD^+^ naïve B cells, confirming the specificity of our staining strategy and demonstrating that all observed antigen-specific populations were vaccine-induced. Crucially, quantitative comparison revealed that the frequencies and absolute numbers of these antigen-specific GC B cells and PCs were substantially higher in mice immunized with AP205 nanoparticles than in those receiving alum-adjuvanted proteins (Figure 5A,B). This indicates a superior capacity of the VLP platform to drive B-cell activation and recruitment into the GC pathway, providing a cellular correlate for the enhanced antibody titers observed in serological assays.

We extended this GC analysis to our advanced bivalent formulations—Chimeric-AP205. Bivalent vaccines potently stimulated GC reactions capable of handling a dual-antigen challenge without evidence of epitope suppression. Flow cytometry revealed the concurrent generation of PCs, GC B cells, and swIg MemB cells specific for both gL/gH and gB (Figure 5C,D). This demonstrates the platform’s robust capacity to deliver complex antigen payloads without compromising the fundamental immunological processes of GC-driven B-cell activation, affinity maturation, and differentiation.

In summary, AP205-based EBV vaccines—in both monovalent and bivalent formats—are potent inducers of high-magnitude, antigen-specific GC responses that are quantitatively superior to those elicited by a standard alum-adjuvanted protein vaccine. These findings provide the critical cellular and mechanistic basis for their superior immunogenicity: the efficient initiation and maintenance of robust GCs directly underpin the rapid onset, high titers, high affinity, and durability of the antibody responses that form the foundation of lasting protective immunity against EBV.

### 3.7. Durability of Neutralizing Antibodies Elicited by EBV gL/gH-, gB- and Chimeric Based VLPs

**Figure 6:**
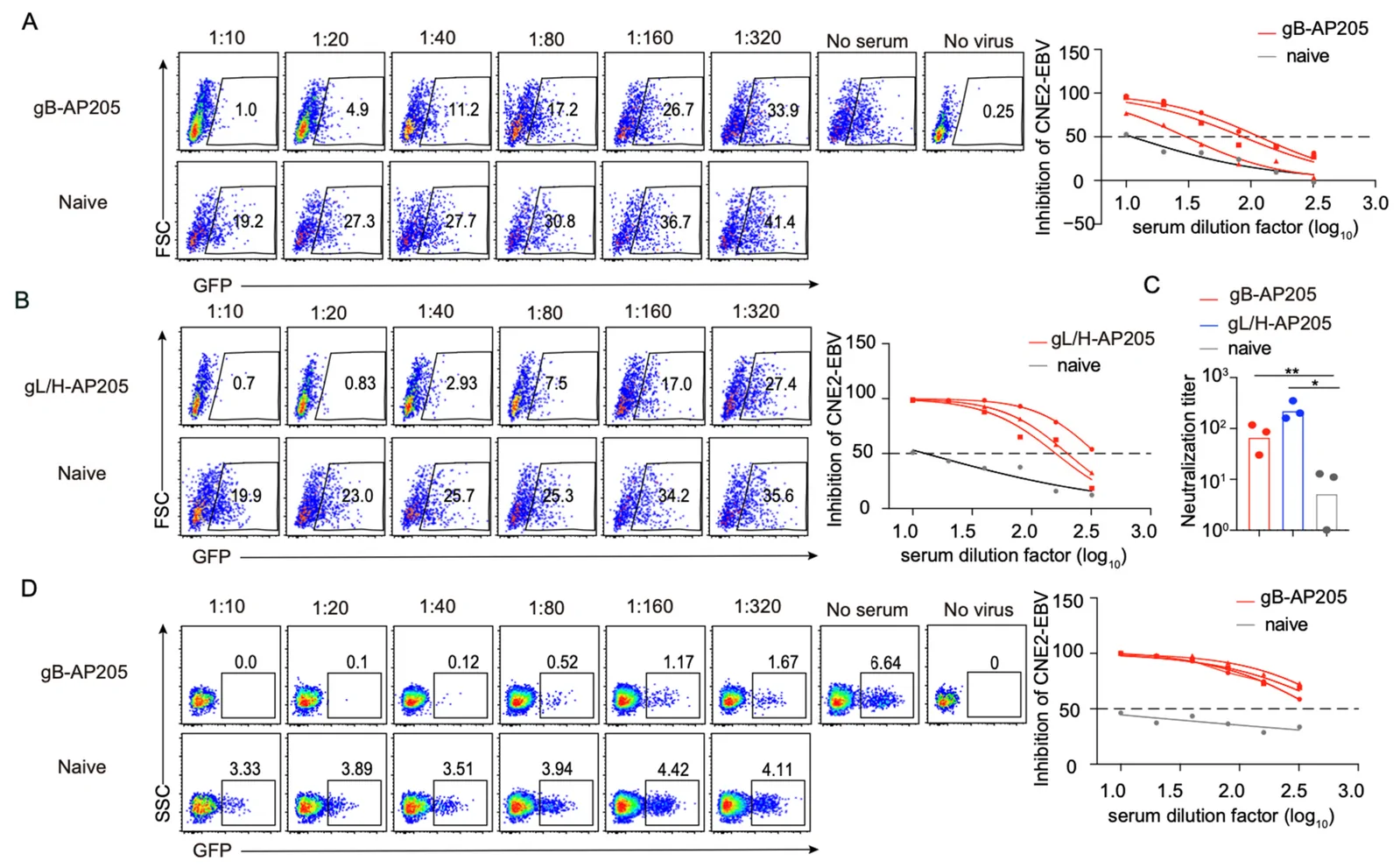

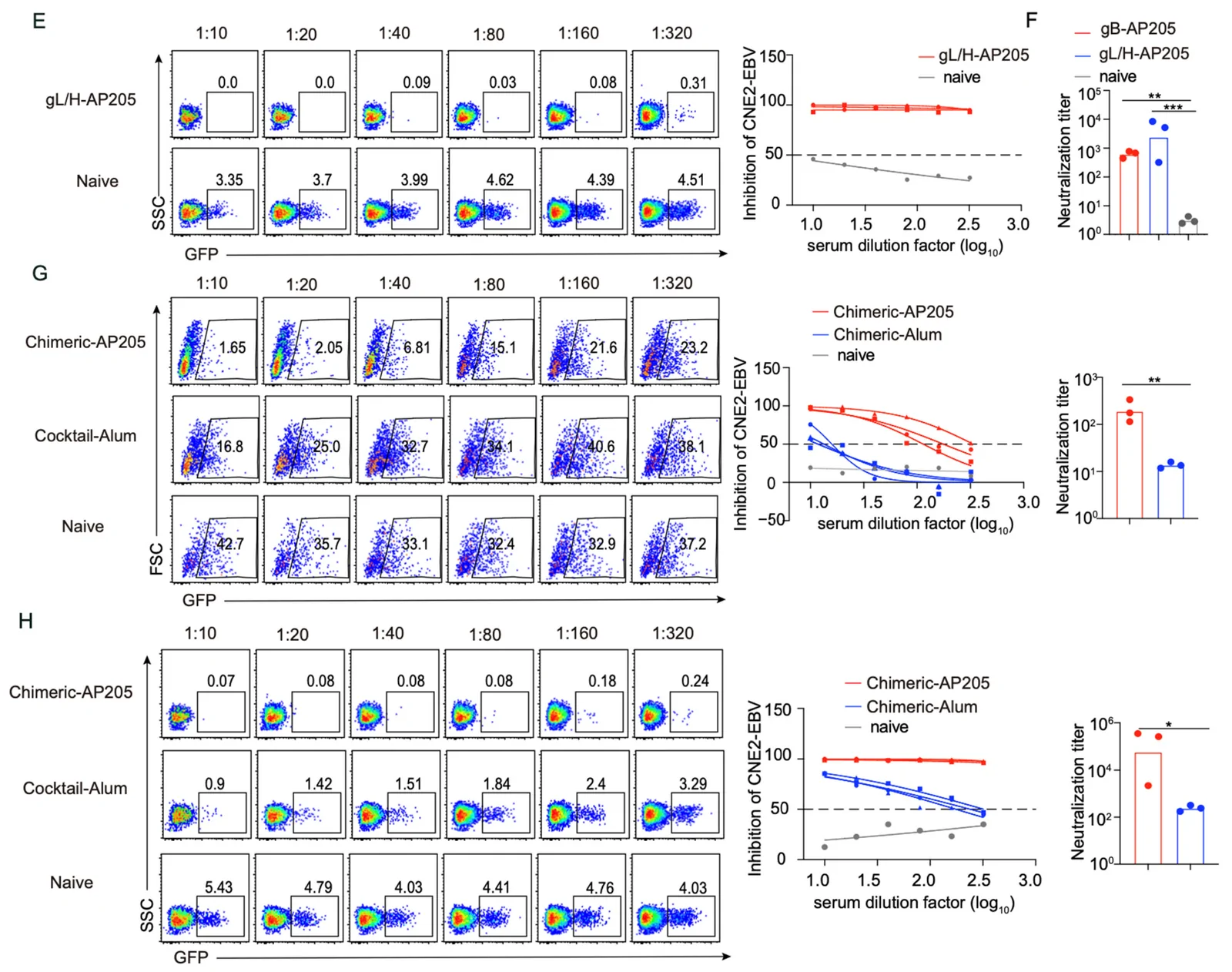
Durability of neutralizing antibodies elicited by EBV gL/gH- and gB-based VLPs versus gL/gH/gB-Alum (*n* = 3). Mice received a single dose of 10 µg of either antigen-AP205, or antigen-Alum via intraperitoneal (i.p.) injection, administered twice with a three-week interval between doses. Then the spleens were analyzed 20 weeks after first immunization. (**A**–**C**) Flow cytometric assessment of the neutralizing activity present in with gB-AP205 (**A**), gL/gH-AP205 (**B**) immunized mice or naive mice sera collected after the second immunization, tested against EBV infection in Raji B cells. Curve fitting was used to determine the half-maximal inhibitory concentrations (IC50), which were presented as the neutralization titers in (**C**). (**D**–**F**) Flow cytometric assessment of the neutralizing activity present in with gB-AP205 (**D**), gL/gH-AP205 (**E**) immunized mice or naive mice sera collected after the second immunization, tested against EBV infection in 293T cells. Curve fitting was used to determine the half-maximal inhibitory concentrations (IC50), which were presented as the neutralization titers in (**F**). (**G**,**H**) Flow cytometric assessment of the neutralizing activity present in with Chimeric-AP205, Chimeric-Alum immunized mice or naive mice sera collected after the second immunization, tested against EBV infection in Raji B cells (**G**), and 293T epithelial cells (**H**). Curve fitting was used to determine the half-maximal inhibitory concentrations (IC50), which were presented as the neutralization titers in the right. one-way ANOVA with Tukey’s post-test was used for C and F; Student’s *t*-test was used for G and H (* *p* < 0.05, ** *p* < 0.01, *** *p* < 0.001).

The long-term efficacy of a prophylactic vaccine relies as heavily on the persistence of protection as on the peak magnitude of the initial response. To assess durability, we collected sera over a four-month period following the final boost and evaluated their neutralizing capacity against both B-cell and epithelial-cell infection. All VLP-based immunogens (monovalent gL/gH-AP205, gB-AP205) elicited potent, broadly reactive antibodies that effectively blocked viral entry into B cells (Figure 6A–C). This protective activity extended equally to epithelial cells—key players in primary infection and viral spread—as evidenced by the marked reduction in infection in these cells (Figure 6D–F).

We further compared the chimeric-AP205 bivalent vaccine against a standard alum-adjuvanted soluble protein regimen. The chimeric formulation consistently outperformed the alum control, achieving superior neutralization titers against both cell types (Figure 6G,H). Collectively, these findings demonstrate that the nanoparticle platform sustains robust cross-neutralization across both major cellular targets of EBV over an extended timeframe.

## 4. Discussion

The development of a prophylactic vaccine against Epstein–Barr virus has been a long-standing and complex challenge, primarily due to the pathogen’s dual cellular tropism and the relatively poor immunogenicity of its essential glycoprotein antigens [15,51,52]. In this study, we have engineered a nanoparticle vaccine platform that directly addresses these hurdles. By employing a rational design that combines antigen multimerization on a virus-like particle scaffold with integrated TLR-mediated adjuvant signaling, we demonstrate that the AP205-based vaccines displaying EBV gL/gH and gB elicit exceptionally potent and durable neutralizing antibody responses capable of blocking infection in both B cells and epithelial cells. 

Despite these advances, a critical knowledge gap remains in the field of EBV VLP vaccines: most existing approaches rely on eukaryotic expression systems and lack systematic comparison of monovalent versus chimeric antigen display. Our study addresses this gap by introducing an AP205-based synthetic VLP platform produced in *E. coli*-a modular, scalable, and intrinsically adjuvanted system that enables precise control over antigen valency and orientation, features that collectively distinguish our platform from previous EBV VLP candidates.

The profound immunogenicity of our platform stems from a synergistic integration of key design principles. First, the repetitive, high-density display of antigens on the AP205 VLP surface efficiently cross-links B cell receptors, driving robust clonal expansion and differentiation—a mechanism that explains the significantly higher antibody titers compared to soluble, alum-adjuvanted protein [33,34,35]. Second, the SpyTag/SpyCatcher system ensures stable, covalent, and oriented antigen presentation [27,36,37], overcoming the instability and heterogeneity of chemical conjugation to produce a well-defined and consistent immunogen. Third, the encapsulated bacterial ssRNA provides an intrinsic TLR7/8 adjuvant signal, promoting a Th1-polarized response as evidenced by elevated IgG2a titers—a profile associated with potent effector functions and superior antiviral immunity compared to the Th2-skewed response typical of alum [28].

It is important to consider whether residual *E. coli*-derived LPS/endotoxin could contribute to the observed immunogenicity. While we did not perform independent LAL testing, the AP205 particles were obtained from a well-validated source, and the platform’s established safety and purification profile support minimal endotoxin carryover. Instead, the enhanced immunogenicity is likely attributable to the high-density antigen display, the intrinsic ssRNA-mediated TLR7/8 activation (a well-characterized property of the AP205 platform), and the particulate nature of the VLP itself [46,53].

Beyond neutralization, the observed IgG2a bias also suggests that our VLP vaccine may elicit more potent Fc-dependent effector functions, including antibody-dependent cellular cytotoxicity (ADCC), antibody-dependent cellular phagocytosis (ADCP), and complement-enhanced neutralization or complement-dependent cytolysis [54,55,56]. These activities have been increasingly recognized as critical correlates of protection against viral infections, including EBV [57]. While we did not directly evaluate these functionalities in the current study, the enhanced IgG2a/IgG1 ratio associated with AP205-based immunization is consistent with a Th1-skewed response that favors the production of antibodies with higher affinity for activating Fcγ receptors. Future studies incorporating ADCC reporter assays, macrophage phagocytosis assays, and complement-mediated neutralization assays will be essential to fully characterize the functional repertoire of antibodies elicited by our VLP platform.

Another important aspect of EBV vaccine development is the induction of cellular immunity, particularly cytolytic CD8^+^ T cell responses, which are critical for controlling EBV persistence and reactivation [57,58,59]. Given the inherent immunostimulatory properties of VLP-based vaccines—including their ability to cross-present antigens to CD8^+^ T cells via MHC class I pathways—it is plausible that our AP205 VLP platform may also stimulate T cell responses [29,60]. While we did not investigate T cell responses in the current proof-of-concept study, the AP205 VLP platform has been shown by others to elicit antigen-specific T cell responses in various vaccine settings. We have now identified this as a priority for future investigation, which could include IFN-γ ELISpot, intracellular cytokine staining, and in vivo cytotoxicity assays to determine whether our vaccine candidates elicit functional cytolytic T cell activity.

Our systematic comparison of vaccine architectures established a clear structure–immunogenicity hierarchy, identifying the bivalent chimeric VLP—which co-displays gL/gH and gB on a single particle—as the most potent candidate. Its advantage over the bivalent cocktail likely stems from spatial co-localization of the two antigens on the same scaffold. This design may facilitate simultaneous uptake and processing by the same antigen-presenting cell, thereby promoting coordinated T-cell help that benefits B cells specific for either glycoprotein. Moreover, presentation of both antigens on a single nanoscale particle may more faithfully recapitulate their native arrangement on the virion surface than a physical mixture of separate particles, potentially eliciting antibodies with superior neutralizing activity against the authentic entry complex.

It is worth noting that, compared to the monotypic VLP groups, the chimeric VLP induced slightly lower ELISA titers for both gL/gH and gB, consistent with the reduced copy number of each antigen when two antigens compete for limited conjugation sites on the same particle. Interestingly, however, the chimeric VLP elicited slightly higher neutralization titers than either monotypic VLP alone. This apparent discrepancy can be explained by the spatial proximity of gL/gH and gB on the same nanoscale scaffold, which may better mimic their native arrangement on the virion surface and promote the generation of antibodies that recognize the authentic entry complex more effectively [61,62]. The reduced antigen density per individual glycoprotein may compromise total binding antibody titers, but the cooperative display of both entry glycoproteins on a single particle likely enhances the functional quality of the neutralizing antibody response [63]. This observation underscores the importance of antigen spatial organization in shaping antibody functionality beyond simple titer measurements, and supports the chimeric VLP as a promising multivalent vaccine candidate.

Despite these promising results, several limitations merit consideration. Our findings derive from a murine model, and the translatability of the observed immune responses to humans awaits confirmation. Future studies in humanized mice or non-human primates capable of supporting full EBV infection will be essential to assess translational potential. In addition, further investigation into the anti-AP205 carrier response and its possible synergy with antigen-specific immunity could inform scaffold optimization. Furthermore, we did not quantitatively assess the relative binding ratio of gL/gH versus gB on the chimeric VLPs; while SDS-PAGE suggested comparable loading levels, the precise occupancy of each antigen remains to be determined. This represents a point for future investigation, potentially using mass spectrometry or quantitative densitometry with antigen-specific standards [61]. Additionally, as noted above, we did not assess Fc-dependent effector functions (ADCC, ADCP, complement) or T cell responses in the current study. While the IgG2a bias provides indirect evidence supporting potential Fc-mediated activity and T cell help, direct experimental confirmation is warranted. We acknowledge these as important limitations and have outlined them as key priorities for future work. Finally, extended longitudinal studies beyond one year are needed to fully characterize the persistence of protective memory.

In summary, we have developed and validated a modular VLP platform that successfully transforms the intrinsically weak immunogenicity of soluble gL/gH and gB into a powerfully immunogenic nanovaccine. This approach overcomes the key obstacles in EBV vaccine development by eliciting broad, potent, and durable neutralization across both major cellular tropisms of the virus. Moreover, the qualitative features of the antibody response—particularly the Th1-biased isotype profile—point toward the potential for robust Fc-mediated effector functions and provide a foundation for future exploration of cellular immunity. More broadly, the AP205-SpyTag/SpyCatcher system exemplifies a generalizable vaccinology strategy, demonstrating that precise nanoscale antigen organization combined with intrinsic adjuvant signaling can be harnessed to program high-quality adaptive immunity [28]. This work represents a meaningful advance toward an EBV vaccine and highlights the translational promise of engineered nanomaterials in preventive medicine.

## 5. Conclusions

In this study, we successfully developed a modular AP205-based VLP platform that overcomes the poor immunogenicity of EBV entry glycoproteins gL/gH and gB through precise nanoscale antigen display. Our head-to-head comparison of vaccine architectures established the bivalent chimeric VLP—co-displaying both antigens on the same particle—as the optimal formulation, eliciting higher neutralizing antibody titers and durable protection against EBV infection in both B cells and epithelial cells. Mechanistically, the platform’s intrinsic TLR7 agonist activity and robust germinal center responses underpinned the sustained humoral immunity observed over four months. These findings confirm that precision nanoscale assembly of viral antigens on a synthetic VLP can program high-magnitude, broad-spectrum, and long-lasting humoral immunity. The AP205-SpyTag system thus represents a versatile and generalizable platform with significant translational potential, not only for EBV but also for rapid adaptation to other viral pathogens. This work provides a strong foundation for future clinical development of a prophylactic EBV vaccine and offers a blueprint for next-generation nanovaccine design.

## Data Availability

The original contributions presented in this study are included in the article. Further inquiries can be directed to the corresponding authors.

## Supplementary Materials

The following supporting information can be downloaded at: https://www.mdpi.com/article/10.3390/vaccines14090735/s1, Figure S1: Original agarose gel of gL/gH-SpyCatcher, gB-SpyCatcher and gL/gH-AP205, gB-AP205 particle.
