# Supplementary material for: Dual Antigen Display on an AP205 VLP Platform Elicits Potent and Durable Neutralization of EBV Infection in B Cells and Epithelial Cells In Vitro
Source: Vaccines (Basel). 2026 Aug 25;14(9):735. doi: 10.3390/vaccines14090735 (PMC13611383; doi:10.3390/vaccines14090735)
Supplement: Supplementary file 1 [file vaccines-14-00735-s001.zip › vaccines-4443583-supplementary.pdf]

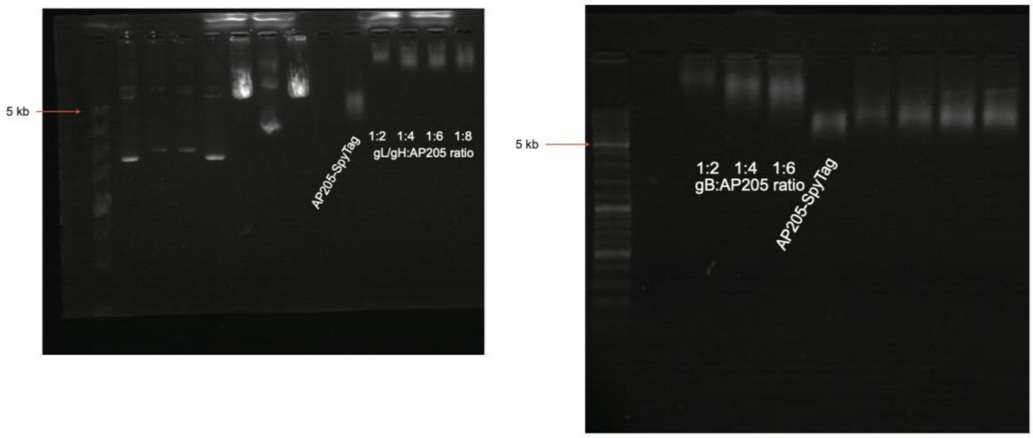

Figure S1: Original agarose gel of gL/gH-SpyCatcher, gB-SpyCatcher and gL/gH-AP205, gB-AP205 particle.
